# Signature on consent form when the patient's right/left hand is impaired

**Published:** 2009-02

## Facts of the case

- The patient was admitted with a complaint of stomach pain and underwent “appendicectomy”. Thereafter, the patient developed complications such as a high blood urea volume (BUN) and s. creatinine along with hematemisis, and hematuria. He was transferred to another hospital where he died.

## Patient's allegation/s of medical negligence

- It was alleged that consent of the patient was not taken to conduct the surgery.
- It was alleged that the surgeon (OP) did not conduct pre-operative investigations such as a complete hemogram, in general and blood sugar, clotting time (CT), and bleeding time (BT), in particular.

## Doctor's defense

- It was stated by the surgeon (OP) in defense that as an IV line had been secured on the right hand of the patient, the patient told the surgeon to take signature of one of his close friends, who had accompanied the patient.
- It was stated by the surgeon (OP) that he did not think it necessary to do a complete hemogram, blood sugar, and tests like CT and BT as the patient had no previous history suggestive of renal disorders, diabetes or bleeding disorders.

## Findings of the court

- The court held that though the patient was in a position to give consent, the printed consent form was signed by someone who had accompanied the patient and not even by a blood relative. The court observed that the surgeon's (OP) defense that the patient had an IV line on his right hand was an afterthought, as it could not have come in the way of the patient signing the consent form. Further, the court observed that the surgeon (OP) could have obtained a left thumb impression of the patient on the consent form, and hence, held that there was no consent of the patient to undergo the surgery.
- The court relied on medical literature, which stated that for all surgeries, a hemogram test is an absolute necessity. In fact, another doctor who was examined on behalf of the surgeon (OP) admitted that every operation requires a hemogram investigation. The court held that conducting the procedure without a hemogram amounts to deficiency in service and negligence.
- The court further advised the State Government to lay down guidelines for hospitals.
- Hence, the surgeon (OP) was held negligent.

## Suggested Precautions

1. In case a patient is unable to sign the consent form with right/left hand (in the instant case it was due to an intravenous (IV) line), take thumb impression of the other hand on the consent form. Specifically record the reasons for taking thumb impression on the consent form. In all such cases, it is advisable to take suitable endorsement from the patient's relatives/friends/attendants on the consent form.
2. Discharge summary/ticket must be prepared in duplicate. Acknowledgment of receipt must be taken from the patient or relatives/friends/attendants on duplicate before issuing the original copy.
3. Requisite investigations are mandatory before conducting any surgery/procedure.
4. Medical records of every IPD patient must be maintained carefully – recording each and every step of treatment.

